# Notices, &c.

**Published:** 1859-01

**Authors:** 


### NOTICES, &c.

*The Christ Child;* one of the publications of the General Protestant Episcopal S. S. Union. A Christmas book for children; illustrating by a charming little story, the value of Heaven born charity in connection with those beautiful words of the Saviour, "Inasmuch as ye have done it unto one of the least of these my brethren, ye have done it unto me."

"*Philip and Arthur*," or 'Story of the Chatterton Children,' issued by the same Society; instructive to parents as well as children, giving practical lessons in narrative form, in that most difficult and responsible of all family duties, the rearing of our children; teaching the young impressively, that beginning each day well, is to begin life well, and secures for both, the sunshine of true enjoyment and prosperity; while those who are forgetful of duty and are selfish, bring upon themselves a series of disappointments and heart aches, which make of life a miserable failure.

*Blackwood's Magazine*, $3 a year, has greatly added to the practical interest of its pages for some months past, by a series of popularized scientific articles on physiological subjects —Animal Heat, Respiration, Circulation, &c., &c.

For six months past we have missed from our exchange table that safe and instructive family monthly "*The Home*," of Buffalo. We hope it will come regularly hereafter.

*Godey's Lady's Book* for January may well tempt a practical man or woman to subscribe for it, if the three dollars had to be earned by day's work. Mrs. Skimmilk says, for example that "All those who have lived to any purpose in the world, have lived methodically." There is serious truth in that statement, one by which a young man may select with great certainty, a wife worth having. Had we fallen on one who kept things "in a muss," who never knew where to find a thing when wanted, we really think we should have predestrianated indefinitely, especially as we never failed to find that unmethodical people were always dirty, without tidiness or neatness except for the briefest space. Then there are Dr. Wilson's health items, short, wise, timely. The reading of the "Unexpected Visitor" shows at a glance the striking difference between a helpless and a handy wife, the immeasurable distance between the woman who can make circumstances serve her, and the good-for-nothing creature who is "terribly put out" unless every thing is arranged to her hand by somebody else. Next there is "A Days Journey" by Alice B. Haven, so full of human nature, as to equally instruct, "good and bad." Wonder if "Mrs. Graves" is the only woman who always comes home from a visit or a shopping illnatured and cross? What a devlish quality ill nature is in any body, but in a woman, what depth of degradation!

